# Death Cafés: Death Doulas and Family Communication

**DOI:** 10.3390/bs7020026

**Published:** 2017-04-26

**Authors:** Paula K. Baldwin

**Affiliations:** Department of Communication Studies, Western Oregon University, Monmouth, OR 97361, USA; baldwinp@wou.edu; Tel.: +1-503-838-8065

**Keywords:** Death Café, communication, family communication, end of life, death doulas, death

## Abstract

The Death Café is part of the Death Positive movement, and as such, is uniquely positioned to bring the dialogue about death and dying to the public. Participants in a Death Café typically have two different perspectives. Some participants have not experienced death in their family and friends’ circle and wish to converse with others about their beliefs on death and dying. Others are those who have experienced death somewhere in their circle of friends and families. One of goals of the Death Café facilitators is to help attendees reconcile their family narratives regarding death using the broader lens of the Death Café. Using the insights provided by interviews from 15 Death Café facilitators, this manuscript discusses the role of the Death Café facilitators as the death doulas of family communication.

## 1. Introduction

The experience of death in contemporary American society is frequently divided along racial, ethnic, and religious lines [1]. Although the American death culture cannot be categorized easily, American individualism, our unwillingness to accept aging and death, and the “keep them alive at all costs” medical system has a very strong influence on our perception of end of life [2].

Death Café (DC), the Death Salon, and the Order of the Good Death are all part of the Death Positive movement that seeks to counter a collective reluctance to embrace mortality [3,4]. The Order of the Good Death and its event, Death Salon, focus on bringing together professionals to discuss the broader cultural impacts of death; DC focuses on one’s personal interactions with mortality. Death Café is a grassroots organization driven by volunteers who feel strongly about creating a safe space for people to meet, eat cake, drink tea or coffee, and discuss death with no agenda, objectives or themes [5]. Rather than a grief support or counseling session, DC is a private, group-directed discussion.

### Death Cafés

Jon Underwood and Sue Barsky Reid developed the Death Café model based on the ideas of Bernard Crettaz, Swiss sociologist [3]. DC’s objective is to “increase awareness of death with a view to helping people make the most of their (finite) lives” [3,4]. Death Cafes spread quickly across Europe, North America and Australasia (Australia, New Zealand, the island of New Guinea, and neighboring islands in the Pacific Ocean). As of this writing, 4096 Death Cafes in 42 countries have been held since September 2011, all run by volunteers [4].

Holding a DC requires a host and facilitator, a venue with refreshments, and people who want to talk about death. A host is not necessary to the process, but the facilitator is essential. Both roles can be combined, but the facilitator only performs their role during the actual DC session. The DC facilitators (DCF) welcome the attendees to the DC and share the guidelines. Anyone can host a DC, but the DC facilitators and the DC attendees (DCA) must agree to follow the DC guidelines: not-for-profit, held in an accessible, respectful, and confidential space, with no intention of leading people to any conclusion, product, or course of action, and serving refreshing drinks and cake [6]. Rarely do the DCA violate these rules, but if they do, the DCA are reminded of the rules and then redirected. If the attendee chooses not to comply, then they would be asked to leave.

Becoming a DCF is informal. The founders of the DC movement ask that a potential DCF read and download their DC Guide, which includes suggestions for holding a DC [5,6]. They ask that potential facilitators read and adhere to the DC guidelines and post their DC date and information on the DC website.

The DC is an interesting phenomenon to study because the organization is entirely run by volunteers. These volunteers find and reserve the venue, design, print and distribute the fliers, purchase the refreshments, run the DC session, debrief the participants, and clean up afterwards. Many of the DCF hold their DC at least once a month, if not more frequently.

Death Café attendees typically have two different perspectives. Some attendees have not yet experienced death in their family and friends’ circle and wish to converse with others about their beliefs on death and dying. Other DC attendees are those who have experienced the death of a loved one and are driven to make sense of the experience, particularly if the attendee has had a less than satisfying experience with a loved one’s death. Our families are the initial sources for all our beliefs, including our attitudes and opinions, about death and dying [7]. Either directly or indirectly, to some degree, most of the DC attendees have a common goal of reconciling their family narratives regarding death during a Death Café where talking about death is welcomed. As a comparatively new phenomenon, there is relatively little existing academic research on this topic [8,9]. To date, the current scholarship on Death Café focuses on its history and the lack of diversity among attendees, but they do not tell us about the volunteers, the DCF, and why they do this work. Therefore, the purpose of this study was to examine the DCF motivations for holding DC.

## 2. Methods

In order to begin to understand DCF’s motivations for volunteering, interviews were selected as the primary means of data collection. Semi-structured interviews allow the researcher to ask the same basic questions of all interviewees, yet allow for flexibility if topics arise that require additional probing or inquiry. Interviews are also beneficial because they allow participants to reflect upon and make sense of their experiences [10]. In addition to investigating motivations, interview questions further attempted to understand the DCF through a close examination of their personal goals in holding DC, and their observations about themselves and the DCA (see Appendix A for interview questions).

After receiving approval from the author’s institutional review board, participants were recruited through the Death Café website with permission from its founder. A second recruitment solicitation was also sent by email inviting a select group of DCF considered particularly active by having hosted at least six DCs per year. Eligibility criteria also required all participants to be age 18 years or older and able to speak English. A total of 15 facilitators were recruited from the United States (14) and Italy (1). Phone interviews were digitally recorded, transcribed verbatim, and analyzed, resulting in 176 pages of data. To protect the confidentiality of the DCFs, all identifying information was removed from the data presented here. Interviews were conducted until thematic saturation was reached and no new information was found.

Death Café facilitator transcripts were reviewed and analyzed using an iterative process of thematic analysis [11]. The author trained two upper division undergraduate students in the process of coding, using an emergent strategy to identify themes. An intercoder reliability of 92% was reached on approximately 20% of the data. At the conclusion of the coding, the resulting themes were shared with two DCF interviewees for membership check validity, resulting in positive confirmation of the themes. Open coding was used to identify comments suggesting themes, and association into more compact categories reduced to three themes related to family communication about dying and death and the role of the Death Café facilitator in helping DC participants make sense of their musings about mortality.

## 3. Results

The majority of the DCF were ages 55–64 (67%). The DCF were White Non-Hispanic (100%), female (93%), and college educated (80%). Sixty-seven per cent of the DCF were married or in a committed partnership (Table 1 shows a summary of DCF characteristics). Demographic information was not collected about the DCF nuclear or extended family, unless that information was shared during the interview process. The following sections include exemplars of the study themes of advocacy, validation, and personal identity, which illuminate the DCF’s motivations for doing this work.

Table 2 shows the three themes, how they were defined and their saturation.

Nearly 80% of the DCF interviewed work or have worked in some aspect of the death industry, in funeral homes, as grief counselors, social workers, and thanatologists to name a few. That such a large percentage of the DCF interviewed for this study are members of the death industry suggests that they are exceptionally sensitive to the impact that little to no communication has on the surviving family members. As one facilitator remarked,

I work in a cemetery; I am a family service advisor. And I know everyday I’ve met people who have things they want to say but they never get to. Or they don’t have somebody at home to talk with until devastating happens and then they are having to have to deal with grief and that they don’t totally understand the information. So I saw a need for it. I think people just need to talk some of these things out in a non-bereavement setting and since I work with death everyday, it just felt very natural for me to do this. I’m also a cancer survivor and I had to face death at 40. And it made me realize, it just doesn’t escape anybody (T6).

Another DCF remarked: “The goal is really just creating this environment where more people can turn to because they don’t feel comfortable talking to their friends and family…And the goal is really to just keep the conversation flowing and make more and more people aware of what DC is.” (T7) Perhaps having witnessed so much pain and discomfort around end-of-life events, this is one of the sources of the advocacy noted among the study interviewees.

### 3.1. Advocacy

Raising awareness, education, and promotion of change in people’s abilities to communicate openly about issues relating to death and dying comprise the theme of advocacy. All 15 of the DCF commented on their strong commitment to changing the narrative about death and dying by also encouraging the Death Café attendees to continue the communication process with their families. As one DCF says, “Conversation in that DC setting enables them to go to their families and have the kind of conversation they need to have about death, that’s why we have DC.” (T8).

The DCF recognize that expanding the conversation about death is critical in a time when people often actively avoid talking about death until that conversation is thrust upon them. One DCF put it thus:

My goal is to bring that conversation that we have at our DC at each table to bring the conversation home to their table. This is something that needs to be discussed rather than ignored because it is inevitable that we are all going to experience multiple losses, not only our loved ones, but our friends! We just can’t ignore it anymore (T3).

Having experienced death and dying within their own families, and understanding the pain of loss, the DCF are fiercely committed to DC and recognize their role in shaping participants, but also influencing the broader culture that frequently denies the inevitability of death: “I feel like it is important to change, or be an agent of change and I feel like with DC that fits in…changing society’s views on death and dying and bringing that back into everyday life” (T1).

By taking on the role of coordinating and facilitating a DC, DCF take particular ownership or responsibility for advocating or shaping the culture. They also recognize that communication during a DC affirms the concerns that others have about living in a society unwilling to openly discuss dying and mortality.

### 3.2. Validation

Validation for the DCF is about both, the DCF and the attendees, with the larger view of bonding with the participants while building communities within communities. Some DCF had experienced horrific death experiences within their own families. As one facilitator commented, “I did not want people to go through that and [for] people to have those terrible deaths (her family’s experiences with uncle)” (T1). Other DCF recognized that DC offers safe spaces for the attendees: “I think it is an opportunity and a place for people to talk about certain subjects where they don’t have these opportunities in their social lives, or with their families” (T4). DC offers the DCF the outlets to experience validation of the time and effort they expend:

The people. The people just amaze me and the ones that come back and the new ones amaze me and their stories amaze me and I always see that we have a need to come and they have much to say and then I feel like we are providing a service for them and it’s my only way in life to give back, and I’ve been blessed to still be here and its ok to do that (T6).

One of the rewards for the DCF is when the attendees come back with feedback on the positive effect that the DC experience had for their family. A DCA approached one DCF and said, “I just want you to know that I have, with my family, sat down last week and we worked out our will. We did it with our entire family and it’s because of the DC that I had the courage to do that and I want to thank you” (T11).

The same DCF also said, “I have had a mother, a daughter, and an aunt all come to the DC and they came up to me afterwards and they said, ‘You have done such a service for our family’” (T11). Sometimes, it is the DCF family that directly benefits from the DCF participation: “Well it was interesting the first one I had my parents came to, and afterwards they said, ‘Okay come over. We are going to show you where all the papers are.’ And so that was an a-ha for me that it did open within my own family a sharing of information that might not have happened otherwise” (T8).

Serving as a DCF allows both attendees and the facilitator to validate their concerns, while creating opportunities for initiating dialogue with families. This validation contributes positively to DCF’s personal identity formation, which is the third and final theme.

### 3.3. Personal Identity

Identity is confirmed and reinforced for the DCF through what they perceive to be their calling and their personal growth. As one DCF put it,

I benefit a lot from it, from you listening to the conversations and participating in the conversations, and you know there is a superstition that if you talk about death you invite it close…. I feel like talking about death and being part of the DC makes me more alive, it makes me feel very alive and that’s very rewarding and that’s why I keep doing it (T1).

Oftentimes, DC have repeat attendees and they offer the DCF important feedback:

Repeat attendees come back, with things that they tell me they are doing. ‘I got my directive I talked to my husband, daughter about directives.’ They come back like they are giving me gifts of what they have done, like you would with your favorite teacher and I figured if that helps make the process easier, that’s fine (T10).

Many of the DCF view this as a calling:

My goal is to be of service. And it’s a way for me to give back to my community and I think I was probably looking for a way. I see a need in people to search for more meaning, to search for answers. It’s kind of a spiritual thing to me (T6).

Furthermore, as another interviewee said: “We celebrate life, and death is part of that. It’s the end game of everybody’s path. I began to realize so many people didn’t want to talk about it or they didn’t know what to do when the death occurred” (T10). One DCF explains it: “I think a way to really help the planet and the people who are living on it who will die is have them explore this topic with each other, especially their families” (T11) and as another DCF says about holding a DC: “That’s my purpose; it’s my joy” (T10).

## 4. Discussion

The three themes from interviews with DCF indicate that family plays an important role in communication about dying and death. The first place where many learn about death and develop related attitudes and beliefs is in the family, therefore, it is important to consider communication in this context [12]. Bereavement following the loss of a family member can help or hinder family communication. Our families may also make our healthcare and after death care decisions. Thus, attending and participating in a DC can help minimize the silence surrounding mortality and mitigate or even prevent negative reactions.

DCF consistently reported that DCs were helpful to initiating family communication about dying. “What I say at the end of every DC is to bring that conversation that we have at our DC at each table to bring the conversation home to their table” (T3). Furthermore, in most cases, it was the DCFs’ own high levels of personal experience with death, either through work or deaths in their families, which seem to be the impetus for their volunteerism with DC. It is reasonable then, that interviewees would talk about the ways in which they and the conversations they facilitated influenced DC attendee’s family communication. Like the doulas that help birth new lives into the world, the DCF breathe new life into our communication about death and dying into the world; the DCF have become the doulas of death communication. One DCF recognized and remarked: “I feel there is just another kind of honoring the dead in sincere sadness without the regret because I have had the opportunity to dialogue. Nothing is left undone. And people can start really embracing death the way we do birth you know” (T10).

## 5. Conclusions

Death Café seeks to disrupt the proscription around death talk. This study specifically seeks to understand what motivates Death Café facilitators to volunteer. One DCF said it best: “based on my own experience, I really believe that if we speak about death, that we will ease the suffering of those we leave behind” (T3) and through that communication, the DCF and the DC continue to fulfill their personal mission of continuing the conversations around death and dying. Future directions include continuing research on this movement and its ability to shape our end-of-life communication.

## Figures and Tables

**Table 1:** Death Café (DC) Facilitator Characteristics.

Characteristic	*n* = 15 (%)
**Age**	
24–34	1 (7%)
35–44	1 (7%)
45–54	3 (20%)
55–64	10 (67%) *
**Sex**	
Female	14 (93%)
Male	1 (7%)
**Hispanic Ethnicity**	
No	15 (100%)
**Education**	
Some College	2 (13%)
Bachelor	6 (40%)
Master	4 (27%)
PhD	2 (13%)
No Answer	1 (7%)
**Relationship Status**	
Single	2 (13%)
Divorced	1 (7%)
Married	9 (60%)
Committed Partnership	1 (7%)
Widowed	2 (13%)
**Employment**	
Unemployed	1 (7%)
Employed	5 (33%)
Self-employed	5 (33%)
Semi-retired	1 (7%)
Retired	2 (13%)
Student	1 (7%)
**Religious Affiliation**	
Agnostic	1 (7%)
Buddhist	2 (13%)
Spiritual	5 (33%)
Jewish	3 (20%)
Wiccan	1 (7%)
Did not identify	1 (7%)
None	2 (13%)
**Employment and Experience with Death**	
Work(ed) in the death industry (e.g., social workers, hospice volunteers, thanatologists, hospice nurses, funeral directors, hospice social workers)	12 (80%)
Experienced multiple deaths in family and friends	9 (60%)
DC facilitators (DCF) who work(ed) in the death industry and experienced multiple deaths in family and friends	12 (80%)

Note: Percentage total varies due to rounding.

**Table 2:** Thematic Information.

Theme	Definition	Data Counts (Instances Where DCF Mentioned a Theme)
Advocacy	Raising Awareness, Educating, Changing the Narrative	202
Validation	Affirmation for themselves and attendees, bonding with the DC attendees (DCA), Building community	201
Personal Identity	Personal growth, personal calling to be of service in this area	144

## Appendix A

### Abbreviations

**Death Cafes: Death and Dying with a Side of Coffee, Tea and Cake**
Interview Questions 1. How long have you been a facilitator? 2. How did you come to get involved in Death Cafes (DC)? - If someone close to them died, ask what the communication about death and dying with/around that person died was like? - How many Death Cafes have you facilitated yourself or assisted? - What is your goal in hosting meetings like this? 3. What have you observed occurring in the Death Cafes? - Are there one or more particular topics that you have noticed occurring regularly? - If so, why do you think that is? - If the person has given more than one DC ask: do you follow the recommended open format or do you set topics ahead of time? - Why did you make that choice? - Have you notice repeat attendees? - If yes, why do you think that is? - Do you use some format to have people write their responses on, such as a blackboard, a blank poster, a book, with questions such as, “Before I die, I want to…” or “To me, death means…?” - Do you have a favorite conversation starter or icebreaker that you use to get the conversations started? - What was the atmosphere like at the beginning and the end of the DC? - Where was your DC located? (Coffee shop, etc.) - What is your favorite a-ha moment from the participants? - What is your personal favorite a-ha moment? 4. How has your involvement in Death Cafes affected your own communication about death and dying? - Do you have an advanced directive? - Did you have an advanced directive before you began hosting DCs? 5. What keeps you doing DC? 6. If you could offer the living one piece of advice about talking about dying, what would it be? 7. Any final thoughts about death, dying, or Death Cafes?
